# Effect of abatacept on the immunogenicity of 23-valent pneumococcal polysaccharide vaccination (PPSV23) in rheumatoid arthritis patients

**DOI:** 10.1186/s13075-015-0863-3

**Published:** 2015-12-10

**Authors:** Kiyoshi Migita, Yukihiro Akeda, Manabu Akazawa, Shigeto Tohma, Fuminori Hirano, Haruko Ideguchi, Hideko Kozuru, Yuka Jiuchi, Ryutaro Matsumura, Eiichi Suematsu, Tomoya Miyamura, Shunsuke Mori, Takahiro Fukui, Yasumori Izumi, Nozomi Iwanaga, Hiroshi Tsutani, Kouichirou Saisyo, Takao Yamanaka, Shiro Ohshima, Naoya Mori, Akinori Matsumori, Koichiro Takahi, Shigeru Yoshizawa, Yojiro Kawabe, Yasuo Suenaga, Tetsuo Ozawa, Norikazu Hamada, Yasuhiro Komiya, Toshihiro Matsui, Hiroshi Furukawa, Kazunori Oishi

**Affiliations:** Japanese National Hospital Organization (NHO) multi-center clinical studies for evidence-based medicine study group: Japanese study of Randomized controlled study for patients with RA using 23-valent pneumococcal polysaccharide vaccine (RA-PPV23), Higashigaoka 2-5-23, Meguro, Tokyo 152-8621 Japan; Research Institute for Microbial Diseases, Osaka University, Yamadaoka 3-1, Suita, Osaka 565-8563 Japan; Department of Public Health and Epidemiology, Meiji Pharmaceutical University, Noshio 2-522-1, Kiyose, Tokyo 204-8588 Japan; Infectious Diseases Surveillance Center, National Institute of Infectious Diseases, Toyama 1-23-1, Shinjuku, Tokyo 162-8640 Japan; Clinical Research Center, NHO Nagasaki Medical Center, Kubara 2-1001-1, Omura, 856-8652 Japan

**Keywords:** Abatacept, Methotrexate, Opsonization index, Rheumatoid arthritis, 23-valent pneumococcal polysaccharide

## Abstract

**Introduction:**

Patients with rheumatoid arthritis (RA) treated with abatacept (ABT) are at increased risk for vaccine-preventable infections. The aim of the present study is to evaluate the humoral response to 23-valent pneumococcal polysaccharide (PPSV23) vaccination in RA patients receiving ABT.

**Methods:**

The immunogenicity study was nested within a randomized, double-blind placebo-controlled study, designed to evaluate the efficacy of the PPSV23. PPSV23 was given to 111 RA patients, who were classified into three groups: RA control (n = 35), methotrexate (MTX) alone (n = 55), and ABT (n = 21). Before and 4–6 weeks after vaccination, we measured the patients’ concentrations of antibodies against pneumococcal serotypes 6B and 23F using an enzyme-linked immunosorbent assay and determined their antibody functionality using a multiplexed opsonophagocytic killing assay, reported as the opsonization index (OI).

**Results:**

The pneumococcal serotype-specific IgG concentrations and OIs were both significantly increased in all treatment groups in response to PPSV23 vaccination. In the ABT group, the IgG responses for the 6B serotype were lower compared with those in the MTX alone or control groups, whereas the OI responses were similar to those in the other two groups. In a subgroup analysis, the pneumococcal serotype-specific IgG responses were significantly lower in both serotypes (6B and 23F) in the ABT/MTX group; however, the OI responses in the ABT group were not different from the control group. There was no association between the pneumococcal serotype-specific IgG and OI responses for the 6B serotype in patients receiving ABT in contrast to the control or MTX alone patients. No severe adverse effects were observed in any of the treatment groups.

**Conclusions:**

OI responses indicate antibody functionality rather than simply their amount, so the similarity of these measurements between all three groups suggests that RA patients receiving ABT still benefit from receiving the PPSV23 vaccination, even though they produce less IgG in response to it. The results suggest an influence of ABT on the humoral response to PPSV23 vaccination under MTX treatment; however, preserved opsonin responses are expected in RA patients treated with ABT plus MTX.

**Trial registration:**

University Hospital Medical Information Network Clinical Trials Registry: UMIN000009566. Registered 12 December 2012.

## Introduction

Patients with autoimmune rheumatic diseases are more susceptible to infectious complications during the course of their diseases. Rheumatoid arthritis (RA) treatment can induce immunosuppression and increase the risk of infection [1]. The introduction of biologics has been a major achievement in treating these diseases, but an increased risk of infection associated with these therapies has become evident [2]. Some infections can be prevented by vaccination, which if used appropriately will decrease the burden of infection [3]. It is important to determine if RA patients receiving biologics have normal responses to vaccines.

Abatacept (ABT) selectively modulates the CD80/CD86:CD28 co-stimulatory signal required for full T-cell activation [4]. The efficacy of ABT has previously been demonstrated both in RA patients with an inadequate response to methotrexate (MTX) and in RA patients with an inadequate response to anti-tumor necrosis factor (TNF) therapy [5], and ABT has been approved for the treatment of RA in a number of countries, including Japan. The impact of ABT on humoral responses to T-cell-dependent antigens, such as bacteriophage X174 and keyhole limpet hemocyanin, was previously evaluated in patients treated with ABT, and the responses to these antigens were reduced [6].

Polysaccharides are able to elicit immune responses in the absence of T-cell help, although the magnitude of the response can be marginally affected by immunosuppressive treatments [7]. Recent studies with pneumococcal polysaccharide vaccines with a limited number of RA patients were performed, without control groups, suggesting an adequate response [8]. Regarding the pneumococcal vaccine, the polysaccharide and less T cell-dependent nature of the antigen [9], may account for the preserved immune response during costimulatory modulation with ABT. The results from the earlier report would be strengthened by an inclusion of the relevant control groups because it is crucial to have a group of age-matched RA patients under treatment with MTX alone for comparison [10]. The objective of this study was to investigate the 23-valent pneumococcal polysaccharide vaccine (PPSV23) in patients with established RA who were being treated with ABT alone or in combination with MTX.

## Methods

### Study design and patient population

**Fig. 1:**
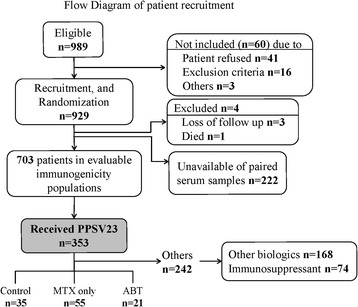
Flow diagram of patient recruitment

This immunogenicity study was nested within a randomized, double-blind, controlled trial designed to evaluate the effectiveness of the PPSV23 in reducing the incidence of pneumonia as a primary endpoint. Patients with clinically diagnosed RA were recruited in Japanese National Hospital Organization (NHO) hospitals across Japan (n = 32) from September 2010 to December 2012 [11]. A total of 932 RA patients were enrolled and randomized 1:1 to receive either the PPSV23 or placebo. Of these, paired serum samples were obtained before and after vaccination from 703 patients, 353 of which received PPSV23. Among these 353 patients,121 patients receiving disease-modifying anti-rheumatic drugs (DMARDs), MTX, or ABT with/without MTX were subjected to the nested study for vaccine immunogenicity (Fig. 1).

Eligible patients were also found to be at risk for developing respiratory infections. RA patients were divided into the following groups: (1) patients with rheumatoid lung disease, (2) patients with RA treated with biological agents, and (3) patients treated with immunosuppressive agents. Patients who had received PPSV23 previously were excluded from this study. This study complies with the principles of the Declaration of Helsinki and was approved by the appropriate institutional review boards at each participating center. All patients provided written informed consent. This study was approved by the ethical committees of NHO central IRB (No. 0512014, 2012) for all participating NHO hospitals and was registered with UMIN-CTR (UMIN000009566).

### Intervention

Patients were randomly assigned to receive either 0.5 ml (25 μg) of PPSV23 (Pneumovax NP, Merck Sharp & Dohme Corp., Tokyo, Japan) or 0.5 ml of a placebo (sodium chloride) subcutaneously in the upper arm. The vaccines were prepared in a masked fashion for those who administered them, blinding both the administrator of the vaccine and the patient to the type of vaccine given. Vaccine and placebo were presented in identical single dose syringes and needle combinations that were labelled with sequential study numbers only. A statistician who was not on the study team carried out the randomization using a random number table and numbered the containers accordingly.

### Enzyme-linked immunosorbent assays for serotype-specific IgG

Blood samples were drawn at vaccination and four to six weeks thereafter, and stocked at −30 °C. Enzyme-linked immunosorbent assays (ELISAs) for serotype-specific IgG were performed to measure the concentration of each type of antibody as previously described [12]. For the measurement of IgG specificity for the 6B and 23F serotypes, we specifically performed our ELISAs according to the World Health Organization (WHO) standard procedure that used the international reference serum, 89SF-3 (graciously supplied by Dr. Carl E. Frasch). To improve the specificity of the assay, a pneumococcal cell wall polysaccharide (C-PS) and pneumococcal 22F polysaccharide pre-absorption step was performed on the samples. The reference serum was pre-absorbed with only C-PS [13, 14]. Detailed protocols are available at www.vaccine.uab.edu/ELISAProtocol(89SF).pdf.

### Multiplexed opsonophagocytic assays

To measure antibody functionality against pneumococcus, we performed multiplexed opsonophagocytic assays (OPAs) for pneumococcal serotypes 6B and 23F, using differentiated HL-60 cells and an antibiotic-resistant target bacteria strain, at the Research Institute for Microbial Disease, Osaka University, as previously described [15]. The quality control serum included in each assay was prepared from pooled sera of adults immunized with PPV23. Opsonization indices (OIs) were defined as the serum dilution that led to 50 % death of target bacteria. Opsotiter 3, an excel-based data processing program, was used to convert colony counts to OIs, according to the WHO protocol available at www.vaccine.uab.edu/UAB-MOPA.pdf.

### Antibody response

Fold increases relative to pre-vaccination values (post-vaccination value to pre-vaccination value ratios) were determined. Positive antibody response was defined as a two-fold or more increase in IgG concentrations or as a ten-fold or more increase in OIs as described previously [12].

### Statistical analysis

The study population was classified into three groups based on the RA treatment at the time of vaccination. Clinical and demographic characteristics of each group were expressed as mean ± standard deviation or as a percentage. Changes in IgG geometric mean concentrations (GMCs) and OPA titers before and after the vaccination were compared using the paired-sample t test. To compare categorical variables in response rates between groups, the Pearson chi-square test was used. Continuous variables were compared using Mann–Whitney tests. For all tests, probability values (*p* values) less than 0.05 were considered statistically significant. All the statistical analyses were performed using the Statistical Analysis System (SAS) and SPSS version 18 software (SPSS, Chicago, IL, USA).

## Results

### Clinical and demographic characteristics

A total of 989 RA patients were assessed for eligibility, and 929 patients were recruited and randomized. Of these, 121 patients receiving disease-modifying anti-rheumatic drugs (DMARDs), MTX, or ABT with/without MTX were subjected to the nested study for vaccine immunogenicity (Fig. 1). The clinical and demographic characteristics of these 121 subjected patients are summarized in Table 1. The study population was classified into three groups: DMARD treatment only (RA control group; n = 35), MTX monotherapy (MTX alone group, n = 55), and ABT treatment (n = 24, mean dose; 547 + 127.9 mg/4 weeks). The mean ages of patients in the ABT group were significantly lower compared to those in the control group. The three groups were otherwise similar. All patients fulfilled the criteria of safety required for vaccine injection, and no serious side effects were observed after vaccination.

**Table 1:** Clinical and demographic characteristics of RA patients prior to pneumococcal vaccination

	RA control	MTX group	ABT group	*p* Values between treatment groups
	n = 35	n = 55	n = 21	
Male/female	12/23	11/44	4/17	0.251
Age, mean ± SD (years)	70.5 ± 10.8	63.8 ± 11.5	59.8 ± 12.0	0.002
Weight, mean ± SD (Kg)	53.3 ± 9.5	52.9 ± 11.8	55.2 ± 10.1	0.501
BMI, mean ± SD	21.8 ± 3.5	21.7 ± 3.7	23.2 ± 4.9	0.344
RA duration, mean ± SD (years)	11.7 ± 12.5	14.1 ± 10.9	13.5 ± 11.2	0.144
MTX dose, mean ± SD (mg/week)	-	7.8 ± 2.4	7.2 ± 3.4(15/21)	-
ABT dose, mean ± SD (mg/4 week)	-	-	547.6 ± 127.9	-
Use of prednisolone, number of patients (%)	21 (60.0)	30 (54.5)	13(61.9)	0.798
Prednisolone dose, mean ± SD (mg/day)	6.06 ± 4.23	4.98 ± 2.97	5.04 ± 2.85	0.769
DAS28(CRP), mean ± SD	2.79 ± 1.17	2.61 ± 0.98	2.48 ± 1.31	0.565
SDAI, mean ± SD	9.03 ± 6.32	8.15 ± 7.33	8.32 ± 7.29	0.593
CDAI, mean ± SD	7.83 ± 5.40	7.46 ± 6.73	7.87 ± 6.54	0.721
IP (%)	6 (17.1)	7 (12.7)	3(14.3)	0.844
COPD (%)	3 (8.6)	1 (1.8)	2(9.5)	0.251
Smoking history (%)	10(28.6)	13(23.6)	6(28.6)	0.839

Data were obtained immediately before pneumococcal vaccination. *p* values between treatment groups were determined using the Kruskal-Wallis test. *p* values were calculated with the chi-square test for qualitative data. *RA* rheumatoid arthritis, *MTX* methotrexate, *ABT* abatacept, BMI body mass index, *DAS28* Disease Activity Score 28, *SDAI* simplified disease activity index, *CDAI* clinical disease activity index, *IP* interstitial pneumonia, *COPD* chronic obstructive pulmonary disease

### Pneumococcal serotype-specific IgG concentrations

To evaluate the effect of ABT treatment on the level of pneumococcal serotype-specific IgG produced following PPS23V vaccination in RA patients, enzyme-linked immunosorbent assays were performed to measure the serotype 6B- and 23F-specific IgG levels in patients from each of the three groups before and after vaccination. The ratios between post- and pre-vaccination antibody concentrations are summarized in Table 2. After vaccination with PPSV23, the geometric mean concentrations (GMCs) of both serotype 6B- and 23F-specific IgG were increased in all groups. However, there were large differences in the fold induction of GMC responses among the groups with regard to treatments; for 6B serotypes, a higher post-GMC was obtained in the control (2.38 times) and MTX alone (1.75 times) groups compared with that in the ABT (1.23 times, no significant increase) group.

**Table 2:** Concentrations of pneumococcal polysaccharide antigen serotype-specific IgG antibodies and opsonization indices in the RA treatment groups before and after 23-valent pneumococcal polysaccharide vaccination

		RA Control	MTX group	ABT group
		n = 35	n = 55	n = 21
IgG GMCs (μg/ml)				
6B	Before	0.84(0.58 to 1.11)	1.42(0.86 to 1.97)	1.12(0.80 to 1.45)
	After	4.05(2.13 to 5.97)*	4.36(2.17 to 6.55)*	2.29(0.76 to 3.83)
	Fold increase	2.38(1.41 to 5.62)	1.75(1.15 to 3.11)	1.41(0.87 to 3.09)
23F	Before	1.17(0.85 to 1.48)	1.79(1.33 to 2.25)	1.22(0.79 to 1.65)
	After	11.61(4.16 to 19.07)*	7.41(4.48 to 10.33)*	4.61(2.95 to 6.27)*
	Fold increase	3.36(1.85 to 9.42)	2.00(1.27 to 5.48)	2.45(1.23 to 7.44)
GM-OIs				
6B	Before	17.24(10.96 to 23.53)	150.79(14.85 to 286.74)	61.55(13.78 to 109.32)
	After	981.15(407.24 to 1555.05)*	584.29(270.29 to 898.28)*	1345.19(383.97 to 2306.41)*
	Fold increase	10.22(1.92 to 79.48)	2.57(1.22 to 22.40)	14.83(2.93 to 163.03)
23F	Before	63.21(−6.79 to 133.20)	52.11(14.04 to 90.18)	138.40(−87.83 to 364.63)
	After	713.49(307.97 to 1119.01)*	724.56(336.93 to 1112.19)*	887.76(172.11 to 1603.42)*
	Fold increase	6.86(2.50 to 27.14)	3.75(1.47 to 38.32)	2.97(1.37 to 76.09)

IgG GMCs and GM-OIs are expressed as the mean (95 % CI). Fold increases are expressed as the median (IQR). Differences between pre- and post-vaccination GMCs of serotype-specific IgG were assessed using a paired-sample t test

*RA* rheumatoid arthritis, *MTX* methotrexate, ABT abatacept, *GMC* geometric mean concentration, *GM-OI* geometric mean opsonization index, *IQR* interquartile range

*p < 0.05 compared with pre-vaccination IgG GMCs or GM-OIs

### Opsonophagocytic killing assays

To determine the antibody functionality in these groups, we performed multiplexed opsonophagocytic killing assays and reported the results as the opsonization index (OI). The post-vaccination OIs increased significantly in all treatment groups. The ratios between pre- and post-vaccination are provided in Table 2. In contrast to the GMC (6B) results, there were no differences in the fold induction of OIs for either serotype (6B or 23F) among the ABT and control or MTX alone groups. In this assay, the ABT group showed an antibody response rate that was equivalent to those in the control and MTX groups.

### Antibody response rates

**Fig. 2:**
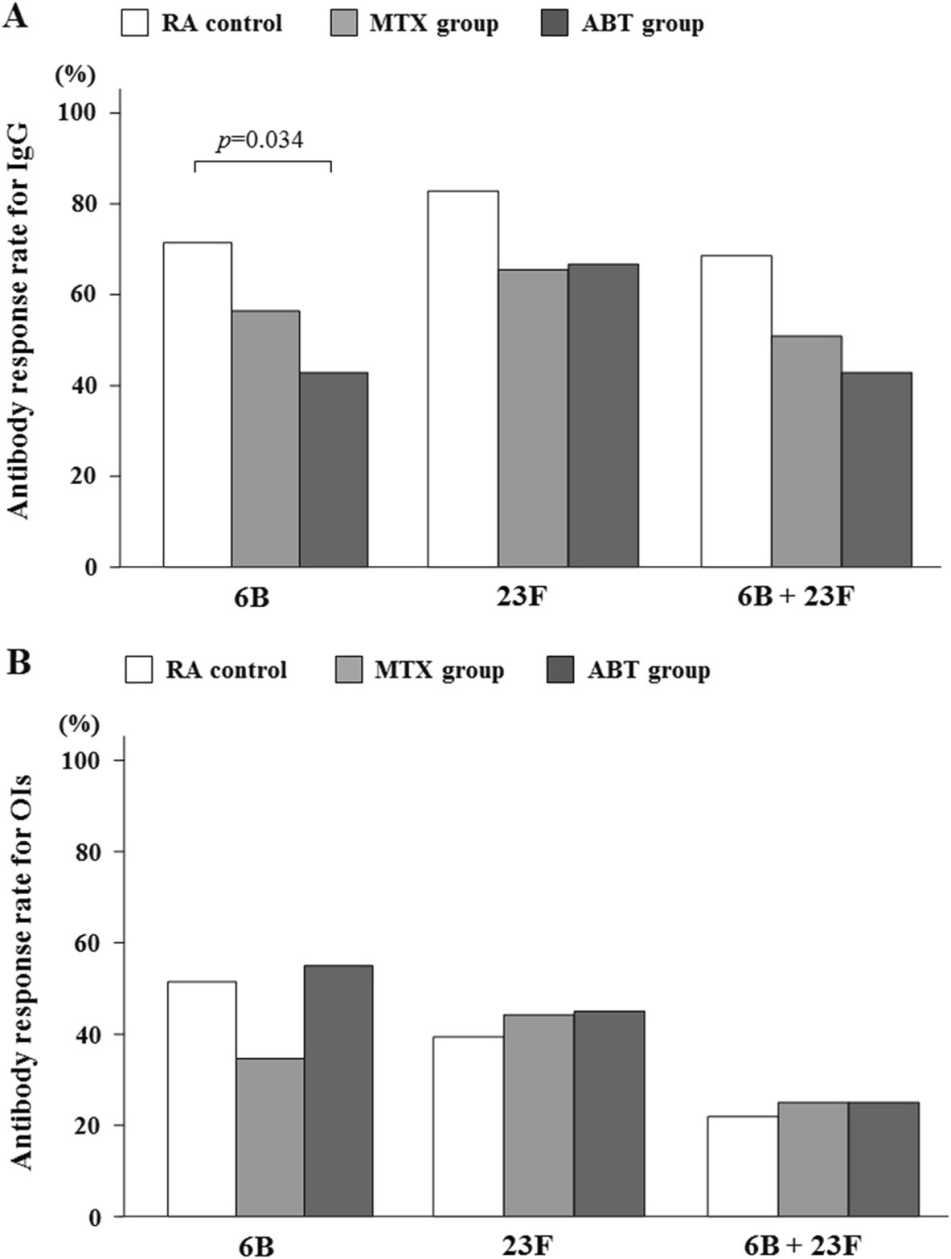
**a** Comparison of post-vaccination GMC responses in patients receiving DMARDs (control), MTX and ABT. Percentages of patients with an increase in 6B or 23F serotype-specific IgG concentration greater than two-fold are shown. There was a significant difference in the 6B serotype-specific IgG response rates between control and ABT groups (*p* = 0.034). Data were compared using the Pearson chi-square test. **b** Comparison of post-vaccination OIs responses in patients receiving DMARDs (control), MTX and ABT. Percentage of patients with an increase in OIs for serotypes 6B or 23F greater than ten-fold are shown. There was no significant difference in the response rates among control, MTX and ABT groups. *GMC* geometric mean concentration, *DMARDS* disease modifying anti-rheumatic drugs, *MTX* methotrexate, *ABT* abatacept, *OIs* opsonization index

The GMC response rates, given as the percentage of patients with a positive antibody response, for patients in the ABT group were significantly decreased compared with those for patients in the control or MTX alone groups for serotype 6B (Fig. 2a). For OIs specific to serotype 6B and 23F, the ABT group showed an equivalent antibody response rate, similarly defined as the percentage of patients with a positive OI response, compared with the control or MTX groups (Fig. 2b).

### Subgroup analysis for patients receiving ABT/MTX combination treatment

**Fig. 3:**
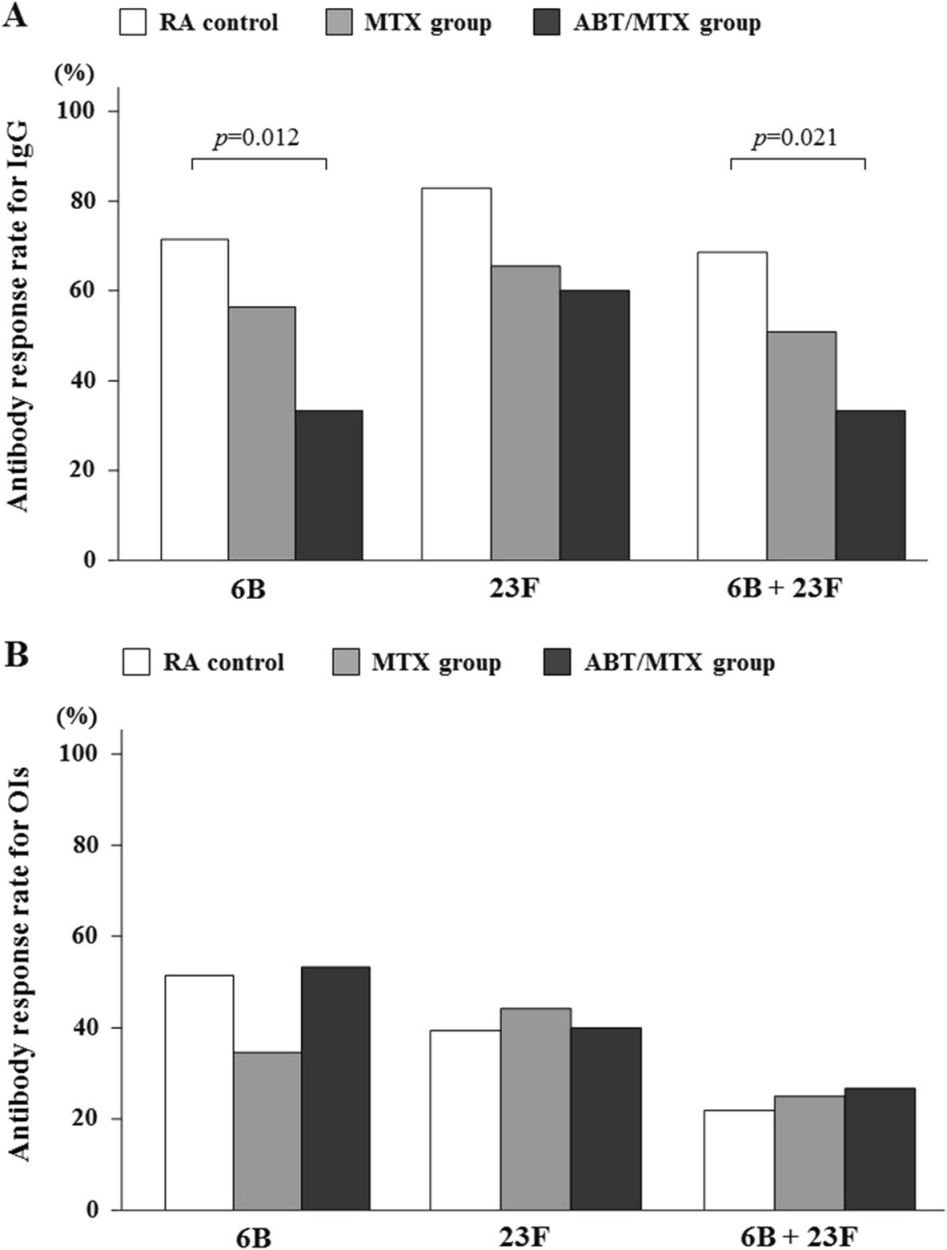
**a** Comparison of post-vaccination GMC responses in patients receiving DMARDs (control), MTX and ABT/MTX. Percentages of patients with an increase in 6B and 23F serotype-specific IgG concentration greater than two-fold are shown. There were significant differences in the 6B and 6B/23F serotype-specific IgG response rates between control and ABT/MTX groups (6B; *p* = 0.012, 6B + 23F; *p* = 0.021). Data were compared using the Pearson chi-square test. **b** Comparison of post-vaccination OI responses in patients receiving DMARDs (control), MTX and ABT/MTX. Percentage of patients with an increase in OIs for serotypes 6B and 23F greater than ten-fold are shown. There was no significant difference in the response rates among control, MTX and ABT/MTX groups. Data were compared using the Pearson chi-square test. *GMC* geometric mean concentration, *DMARDS* disease modifying anti-rheumatic drugs, *MTX* methotrexate, *ABT* abatacept, *OIs* opsonization index

Notably, ABT is primarily used in combination with MTX, and ABT monotherapy is limited in Japan. Therefore, we compared the patients receiving both ABT and MTX (ABT/MTX) to control patients (receiving DMARDs alone or MTX alone). It appears that ABT/MTX groups had an insignificant increase in the GMC for the 6B serotype and an even lower increase in the GMC for the 23F serotype after PPSV23 vaccination compared with control groups (Table 3). However, the post-vaccination OI responses for both serotypes in the patients receiving ABT/MTX combination therapy were equivalent to those in the control group patients. Similarly, a lower proportion of RA patients receiving ABT/MTX had a geometric mean concentration (GMC) increase greater than two-fold for both serotypes; however, the rates of patients with OIs greater than ten-fold for both serotypes were not different between the ABT/MTX and control groups (Fig. 3). Because the opsonophagocytic activity (OPA) is a measurement of antibody function, these results suggest that while the GMC response rate is lower in patients receiving ABT/MTX combination therapy, the antibodies that are produced in response to PPSV23 vaccination by this group are similarly functional.

**Table 3:** Concentrations of pneumococcal polysaccharide antigen serotype-specific IgG antibodies and opsonization indices in the RA treatment groups before and after 23-valent pneumococcal polysaccharide vaccination

		RA Control	MTX group	ABT/MTX group
		n = 35	n = 55	n = 15
IgG GMCs (μg/ml)				
6B	Before	0.84(0.58 to 1.11)	1.42(0.86 to 1.97)	1.19(0.73 to 1.65)
	After	4.05(2.13 to 5.97)*	4.36(2.17 to 6.55)*	2.49(0.29 to 4.70)
	Fold increase	2.38(1.41 to 5.62)	1.75(1.15 to 3.11)	1.19(0.77 to 2.44)
23F	Before	1.17(0.85 to 1.48)	1.79(1.33 to 2.25)	1.17(0.63 to 1.71)
	After	11.61(4.16 to 19.07)*	7.41(4.48 to 10.33)*	3.78(1.87 to 5.70)*
	Fold increase	3.36(1.85 to 9.42)	2.00(1.27 to 5.48)	1.96(1.15 to 5.99)
GM-OIs				
6B	Before	17.24(10.96 to 23.53)	150.79(14.85 to 286.74)	43.33(0.15 to 86.51)
	After	981.15(407.24 to 1555.05)*	584.29(270.29 to 898.28)*	1454.80(109.29 to 2800.31)*
	Fold increase	10.22(1.92 to 79.48)	2.57(1.22 to 22.40)	14.65(2.96 to 193.79)
23F	Before	63.21(−6.79 to 133.20)	52.11(14.04 to 90.18)	178.13(−130.27 to 486.54)
	After	713.49(307.97 to 1119.01)*	724.56(336.93 to 1112.19)*	880.73(−81.54 to 1843.01)*
	Fold increase	6.86(2.50 to 27.14)	3.75(1.47 to 38.32)	2.50(1.33 to 35.74)

IgG GMCs and GM-OIs are expressed as the mean (95 % CI). Fold increases are expressed as the median (IQR). Differences between pre- and post-vaccination GMCs of serotype-specific IgG were assessed using a paired-sample t test

*RA* rheumatoid arthritis, *MTX* methotrexate, *ABT* abatacept, *GMC* geometric mean concentration, *GM-OI* geometric mean opsonization index, *CI*, confidence interval, *IQR* interquartile index

*p < 0.05 compared with pre-vaccination IgG GMCs or GM-OIs

### Associations between pneumococcal serotype-specific IgG and OI responses

**Fig. 4:**
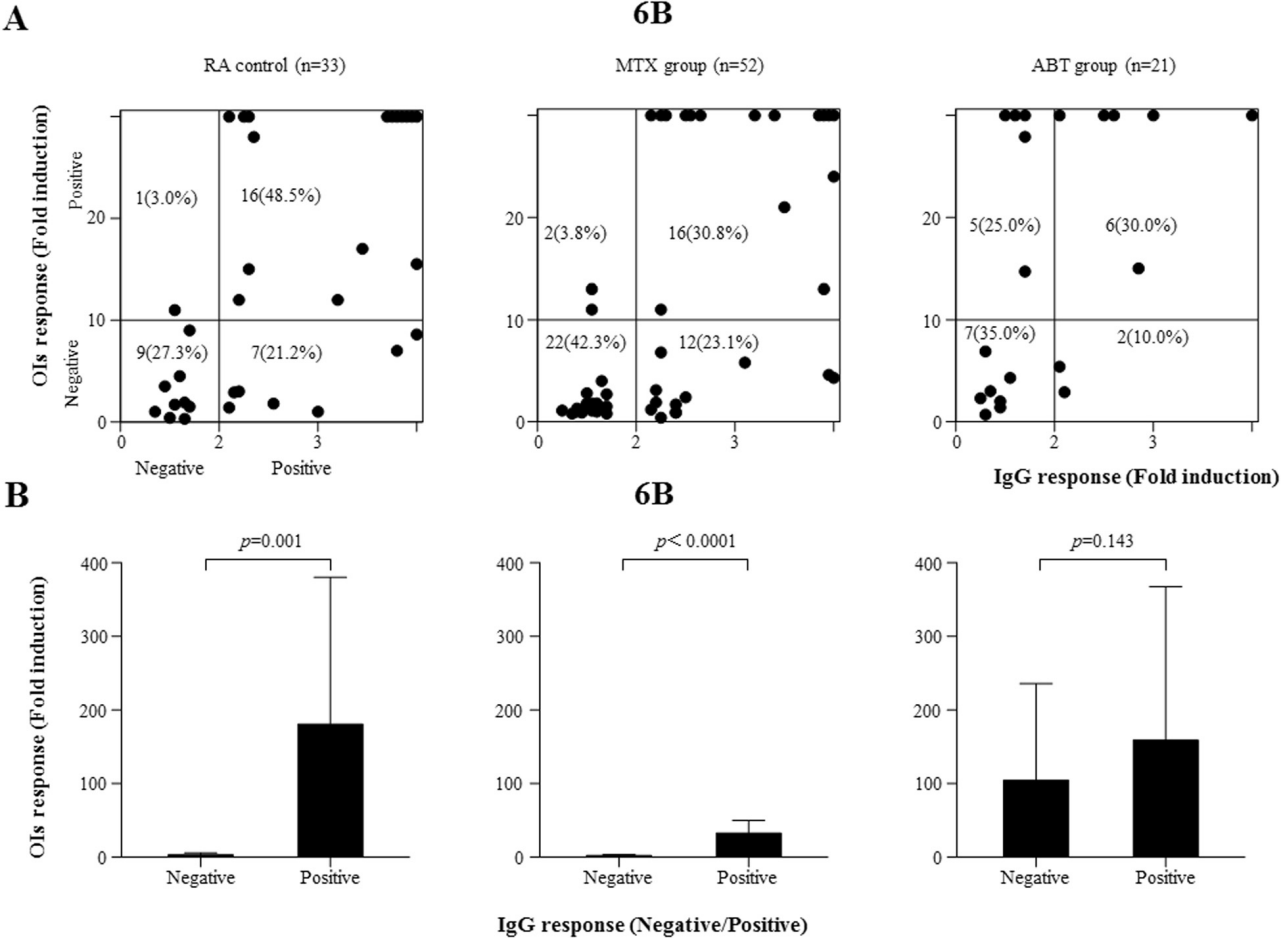
**a** Relationship between IgG and OI responses after PPSV23 vaccination. The 6B serotype-specific IgG (X axis) and OIs (Y axis) responses were plotted in the comparison of three groups (control, MTX, ABT). Positive OI responses were demonstrated in patients receiving ABT with negative IgG responses. **b** Comparisons of means of OIs between patients with negative or positive serotype 6B-specific IgG responses among three treatment groups (control, MTX, ABT). Error bars represent SD of mean OIs. There was no significant difference in mean OIs between patients with or without positive serotype-6B-specific IgG response in ABT group. *IgG* immunoglobulin G, *OIs* opsonization indices, *MTX* methotrexate, *ABT* abatacept, *SD* standard deviation

We assessed the associations between the pneumococcal serotype-specific IgG and OI responses for serotype 6B, in which lower 6B-specific IgG responses were demonstrated in the ABT group. In the control or MTX groups, the functional OI responses were almost exclusively observed in patients who had an optimum pneumococcal serotype-specific IgG response (Fig. 4a). In contrast, in the MTX/ABT group, a positive OI response was also observed in patients who lacked an optimum pneumococcal serotype-specific IgG response (Fig. 4a). The OIs in patients with negative IgG responses were higher in the MTX/ABT group than those in the control or MTX groups (Fig. 4b). Therefore, an association between the pneumococcal serotype-specific IgG response and the OI response was not demonstrated in patients receiving MTX/ABT combination therapy.

### Safety

There were no reported adverse events associated with PPSV23 vaccination in the patients from this study.

## Discussion

This study was nested within a randomized, double-blind, controlled trial designed to evaluate the effectiveness of the PPSV23, and immunogenicity of PPSV23 vaccination in patients receiving ABT was investigated. ABT, a CTLA-4-immunoglobulin-fusion protein, inhibits T cell activation by binding to CD80 and CD86, thus blocking the interaction with CD28 [4]. ABT acts by inhibiting the costimulatory pathway, which is essential for the generation of an immune response to protein and peptide antigens [16]. The requirement of T-cell co-stimulation for B-cell affinity maturation and for the production of high affinity IgG antibodies has potential implications for pneumococcal vaccination [17]. In an open-label, controlled study in healthy subjects, tetanus toxoid vaccine and PPSV23 were each used to assess the impact of ABT on the memory response to a T-cell-dependent protein antigen and to a less T-cell-dependent polysaccharide antigen, respectively [18]. While ABT blunted the immune response (geometric mean titers) to both vaccinations, it did not significantly inhibit the ability of healthy subjects to develop a two-fold response to either vaccine [18]. The reduced antibody response to the T-cell dependent protein tetanus toxoid antigen is consistent with the known ABT modulation of memory T-cell activation, whereas the inhibition of B–T cell help is likely responsible for the reduced antibody response to T cell-independent polysaccharide antigens, such as the pneumococcal vaccine [19]. Kapetanovic *et al.* studied the effect of ABT on the antibody response to PPSV23 in patients with RA (compared with TCZ) and reported marginally diminished antibody responses in the ABT group [8]. Thus, although there is a paucity of data, it appears that ABT is able to blunt the effectiveness of the immune response, but does not significantly inhibit the ability of healthy subjects and patients with RA to develop a clinically significant positive immune response to PPSV23. RA patients frequently use ABT in combination with traditional DMARDs, including MTX [20]. However, MTX was shown to be associated with reduced immune responses to PPSV23 [10]. It is, therefore, crucial to evaluate the immunogenicity of PPSV23 by comparing RA patients treated with ABT with those treated with MTX. Blockage of the CD80/CD86 co-stimulatory molecules by a variety of activated immune cells can regulate the immune responses [21]. This may result in T cell activation and diminished B-cell immunological response as the consequence of inadequate differentiations into plasma cells.

In the present study, the magnitude of the IgG response to PPSV23 was decreased in RA patients treated with ABT. However, the decreased IgG responses to PPSV23 did not affect the OI responses in these patients. This may have resulted from a diminished B cell immune response as a consequence of inadequate stimulation, such as a lack of the T cell help needed for B cell differentiation. However, we also demonstrated that ABT, even with MTX, did not affect the OI responses to PPSV23 vaccination in RA patients. The mechanism by which ABT with or without MTX affects the IgG responses, but not the OI responses against PPSV23, has not been examined in this study. Unlike ELISA results, OPA results are ideal surrogate markers for vaccine efficacy as they mimic the host defense responses [9]. Recent evidence has shown that older adults have a lower capacity to opsonize pneumococci despite normal IgG levels, owing to a lack of anti-pneumococcal IgM antibodies [22]. These poor correlations between opsonic activity and IgG levels have also been shown in patients who are in immunosuppressive states [23]. RA patients receiving ABT/MTX showed a similar dissociation between opsonic activity and levels of IgG against the polysaccharide capsule of pneumococci.

Pneumococcal polysaccharides are T cell-independent antigens. ABT/MTX treatment may partly impair the T cell-independent antibody response that is normally triggered by PPSV23 vaccination because B cells also express CD80 and CD86 [24], and ABT may have some effects on B cell activation in the presence of MTX, as described previously [21]. In contrast, ABT/MTX treatment did not inhibit PPSV23 vaccination-induced OPA. The relative activities of IgG and IgM antibodies may contribute to the preserved OPA in RA patients treated with ABT/MTX as described previously [25]. Strong correlations between ELISA results and OPA results have been observed in many studies [26]; however, no such correlation was found in RA patients receiving ABT. In this setting, OPA has become a useful measure of pneumococcal vaccine immunogenicity. The introduction of pneumococcal conjugate vaccines may expand the options available for protecting RA patients against pneumococcal infections. Abatacept-treated RA patients had decreased antibody response against pneumococcal conjugate vaccines 7 (PCV7) compared to controls and tocilizumab-treated RA patients [8]. Abatacept attenuates activation of T cells by blocking the interaction between CD80/86 and CD28, a co-stimulation signal for T cell activation, which may contribute to the impaired antibody responses after PCV7 vaccination. Whereas another immunogenicity studies comparing PPSV23 and PCV7 revealed that the OPA and geometric antibody titers PCV7 were comparable to those of PPSV23in RA patients [27]. Further immunogenicity studies comparing PPSV23 and PCV13 are needed.

The primary limitation of this study is the relatively small number of RA patients in each group, particularly the group for ABT/MTX combination treatment. Other limitations include the fact that the antibody response is only a surrogate marker of vaccine-induced protection and the inclusion of a number of patients who were treated with ABT in combination with remedies other than MTX. Furthermore, we choose to investigate serotypes 6B and 23F because they are the main causative serotypes of penicillin-resistant pneumococcal pneumonia in Japan [28]. Lastly, the antibody concentrations necessary for protection against invasive pneumococcal disease in adults have not been clearly defined [29]. Strengths of the present study are the standardized blood sampling and the way that the analyses were blinded for demographic and treatment data.

## Conclusions

In conclusion, our results suggest that the T cell co-stimulation modulator, ABT, with or without MTX, has an influence on the humoral responses to PPSV23 vaccination; however, even in patients concomitantly treated with MTX, the opsonization responses against PPSV23 were preserved in RA patients treated with ABT. These data suggest immunization with PPSV23 resulted in a preserved immune response in RA patients treated with ABT.

## Abbreviations

ABT: Abatacept
ELISA: Enzyme-linked immunosorbent assay
GMCs: Geometric mean concentrations
MTX: Methotrexate
OI: Opsonization index
OPA: Opsonophagocytic assay
PPSV23: 23-valent pneumococcal polysaccharide vaccination
RA: Rheumatoid arthritis
TCZ: Tocilizumab
